# Studies of association of *AGPAT6* variants with type 2 diabetes and related metabolic phenotypes in 12,068 Danes

**DOI:** 10.1186/1471-2350-14-113

**Published:** 2013-10-25

**Authors:** Lena Sønder Snogdal, Niels Grarup, Karina Banasik, Mette Wod, Torben Jørgensen, Daniel R Witte, Torsten Lauritzen, Aneta A Nielsen, Ivan Brandslund, Cramer Christensen, Oluf Pedersen, Knud Yderstræde, Henning Beck-Nielsen, Jan Erik Henriksen, Torben Hansen, Kurt Højlund

**Affiliations:** 1 Department of Endocrinology, Diabetes Research Center, Odense University Hospital, Odense, Denmark; 2 Section of Molecular Diabetes & Metabolism, Institute of Clinical Research & Institute of Molecular Medicine, University of Southern Denmark, Odense, Denmark; 3 The Novo Nordisk Foundation Center for Basic Metabolic Research, Faculty of Health and Medical Sciences, University of Copenhagen, Copenhagen, Denmark; 4 Research Centre for Prevention and Health, Glostrup University Hospital, Glostrup, Denmark; 5 Faculty of Health and Medical Sciences, University of Copenhagen, Copenhagen, Denmark; 6 Steno Diabetes Center, Gentofte, Denmark; 7 Department of General Practice, University of Aarhus, Aarhus, Denmark; 8 Department of Clinical Biochemistry, Vejle Hospital, Vejle, Denmark; 9 Institute of Regional Health Research, University of Southern Denmark, Odense, Denmark; 10 Department of Internal Medicine and Endocrinology, Vejle Hospital, Vejle, Denmark; 11 Faculty of Health Sciences, University of Aarhus, Aarhus, Denmark; 12 Hagedorn Research Institute, Gentofte, Denmark

**Keywords:** Type 2 diabetes, Genetics, Insulin resistance, Human, Lipid droplets, *AGPAT*6, GPAT4

## Abstract

**Background:**

Type 2 diabetes, obesity and insulin resistance are characterized by hypertriglyceridemia and ectopic accumulation of lipids in liver and skeletal muscle. *AGPAT6* encodes a novel glycerol-3 phosphate acyltransferase, GPAT4, which catalyzes the first step in the *de novo* triglyceride synthesis. *AGPAT6-*deficient mice show lower weight and resistance to diet- and genetically induced obesity. Here, we examined whether common or low-frequency variants in *AGPAT6* associate with type 2 diabetes or related metabolic traits in a Danish population.

**Methods:**

Eleven variants selected by a candidate gene approach capturing the common and low-frequency variation of *AGPAT6* were genotyped in 12,068 Danes from four study populations of middle-aged individuals. The case–control study involved 4,638 type 2 diabetic and 5,934 glucose-tolerant individuals, while studies of quantitative metabolic traits were performed in 5,645 non-diabetic participants of the Inter99 Study.

**Results:**

None of the eleven *AGPAT6* variants were robustly associated with type 2 diabetes in the Danish case–control study. Moreover, none of the *AGPAT6* variants showed association with measures of obesity (waist circumference and BMI), serum lipid concentrations, fasting or 2-h post-glucose load levels of plasma glucose and serum insulin, or estimated indices of insulin secretion or insulin sensitivity.

**Conclusions:**

Common and low-frequency variants in *AGPAT6* do not significantly associate with type 2 diabetes susceptibility, or influence related phenotypic traits such as obesity, dyslipidemia or indices of insulin sensitivity or insulin secretion in the population studied.

## Background

Lipid droplets are universal cellular organelles that store neutral lipids, such as sterol esters and triglycerides. Lipid droplets are the main reservoir of energy storage, and provide phospholipids for membrane synthesis, and protect cells from the lipotoxic effects of unesterified lipids [1]. Adipose tissue is the main site of lipid storage. However, type 2 diabetes, obesity and insulin resistance are characterized by hypertriglyceridemia and ectopic lipid storage in various non-adipose tissues. Thus, several studies have reported an association between accumulation of intramyocellular triglycerides and insulin resistance [2-5], and the excessive accumulation of lipids in skeletal muscle, liver and heart and a potential lipotoxic effect on beta-cells has been linked to the pathogenesis of insulin resistance and type 2 diabetes [6-8]. Genetic and acquired syndromes of lipodystrophy are characterized by the inability to store lipids in adipose tissues. This causes hypertriglyceridemia and ectopic deposition of lipids in skeletal muscle and liver, and is associated with severe insulin resistance in the absence of obesity [9]. Variants in genes coding for tissue-specific enzymes involved in *de novo* phospholipid and triglyceride biosynthesis or proteins regulating the formation of lipid droplets could potentially disturb the balance between storage of lipids in adipose tissues and ectopic lipid deposition in non-adipose tissues as mentioned above [1,10], and hence contribute to the pathogenesis of insulin resistance and type 2 diabetes.

The first committed step in *de novo* triglyceride synthesis is the acylation of glycerol-3-phosphate leading to the formation of lysophosphatidic acid [10]. This reaction, which has been considered to be rate-limiting, is catalyzed by glycerol-3-phosphate acyltransferases (GPATs), of which four isoforms have been identified in mammals [10]. GPAT4, which is encoded by *AGPAT6*, is the most recently identified GPAT [11,12]. It was initially classified as a acylglycerol-3-phosphate O-acyltransferase (AGPAT), but later studies found it to be the second microsomal GPAT localized to the endoplasmatic reticulum [10,11]. In mice, *AGPAT6* is highly expressed in brown adipose tissue (BAT), white adipose tissue (WAT) and liver [13,14], whereas in humans, *AGPAT6* mRNA seems to be more ubiquitously expressed with the highest expression in testis, and brain, but also in adipose tissues and skeletal muscle [11,15]. Recent studies have reported that microsomal GPAT activity is enhanced by insulin, and that this may involve insulin-stimulated phosphorylation of the microsomal GPAT3 and GPAT4, implying their importance in insulin action on lipogenesis [14]. Studies of *agpat6*−/−knock-out mice have shown that loss of GPAT4 activity causes a 25% reduction in body weight and resistance to both diet- and genetically induced obesity. This was associated with increased energy expenditure, reduced triglyceride storage in BAT and WAT and liver, and subdermal lipodystrophy [10,12,13]. Based on the lessons learned from the *agpat6*-deficient mice, we hypothesized that variation in the human *AGPAT6* may associate with obesity, hypertriglyceridemia, insulin resistance and type 2 diabetes susceptibility.

The aim of the present study was to investigate whether common and low-frequency variation in *AGPAT6*, encoding a novel GPAT enzyme, GPAT4, was associated with type 2 diabetes, obesity, dyslipidemia or indices of insulin resistance and insulin secretion in a large Danish population.

## Methods

*Participants* In total 11 *AGPAT6* variants were genotyped in 12,068 individuals ascertained from five different Danish study groups: 1) The Inter99 cohort, which is a population-based, randomized, non-pharmacological intervention study of middle-aged individuals for the prevention of ischaemic heart disease (n = 6,287) conducted at the Research Centre for Prevention and Health in Glostrup, Copenhagen (ClinicalTrials.gov ID-no: NCT00289237) [16]; 2) type 2 diabetic patients (n = 1,575) from the population-based, ADDITION Denmark screening study cohort (Anglo-Danish-Dutch Study of Intensive Treatment in People with Screen-Detected Diabetes in Primary Care) (ClinicalTrials.gov ID-no: NCT00237548) [17]; 3) unrelated type 2 diabetic patients (n = 1,658) examined at the out-patient clinic at Steno Diabetes Center, Copenhagen, Denmark; 4) a population-based group of unrelated middle-aged individuals (n = 567) also sampled at Steno Diabetes Center, and 5) a sample of type 2 diabetic patients and age-matched non-diabetic control persons enrolled in the former Vejle County (n = 1,981). The type 2 diabetes inclusion criteria in all study groups fulfilled the diagnostic criteria according to the WHO 1999. Clinical characteristics of the five study groups are given in Additional file 1: Table S1. All participants were Danes by self-report and all gave informed written consent before participation. We do not have specific information on ethnicity yet all study participants are of Danish nationality. In other studies of the same study material we have by chip genotyping data looked at population outliers and did not observe ethnical population outliers [18]. The studies were performed in accordance with the principles of the Helsinki Declaration and all studies were approved by the local ethics committees (Ethical Committee of Copenhagen: #KA 98155, #KA95117g, #KA95117gm, #KA94092g, #KA96008; Ethical Committee of Aarhus: #2000183; Ethical Committee of Region of Southern Denmark: #S-20080097).

Type 2 diabetic patients from study groups 1 (n = 328), 2 (n = 1,575), 3 (n = 1,658), 4 (n = 47) and 5 (n = 1,030) were included in the case–control studies. Control individuals had normal fasting glucose according to WHO 1999 criteria [19] (study group 5 (n = 951)) or had normal fasting glucose and normal glucose tolerance during a standard 75 g OGTT (study groups 1 (n = 4,463) and 4 (n = 520)). No pre-diabetics were included in the study. In total, the case–control studies included 4,638 type 2 diabetic and 5,934 control individuals. Studies of quantitative traits were performed in 5,645 participants of the Inter99 cohort excluding individuals with type 2 diabetes and screen-detected type 2 diabetes.

**Figure 1:**
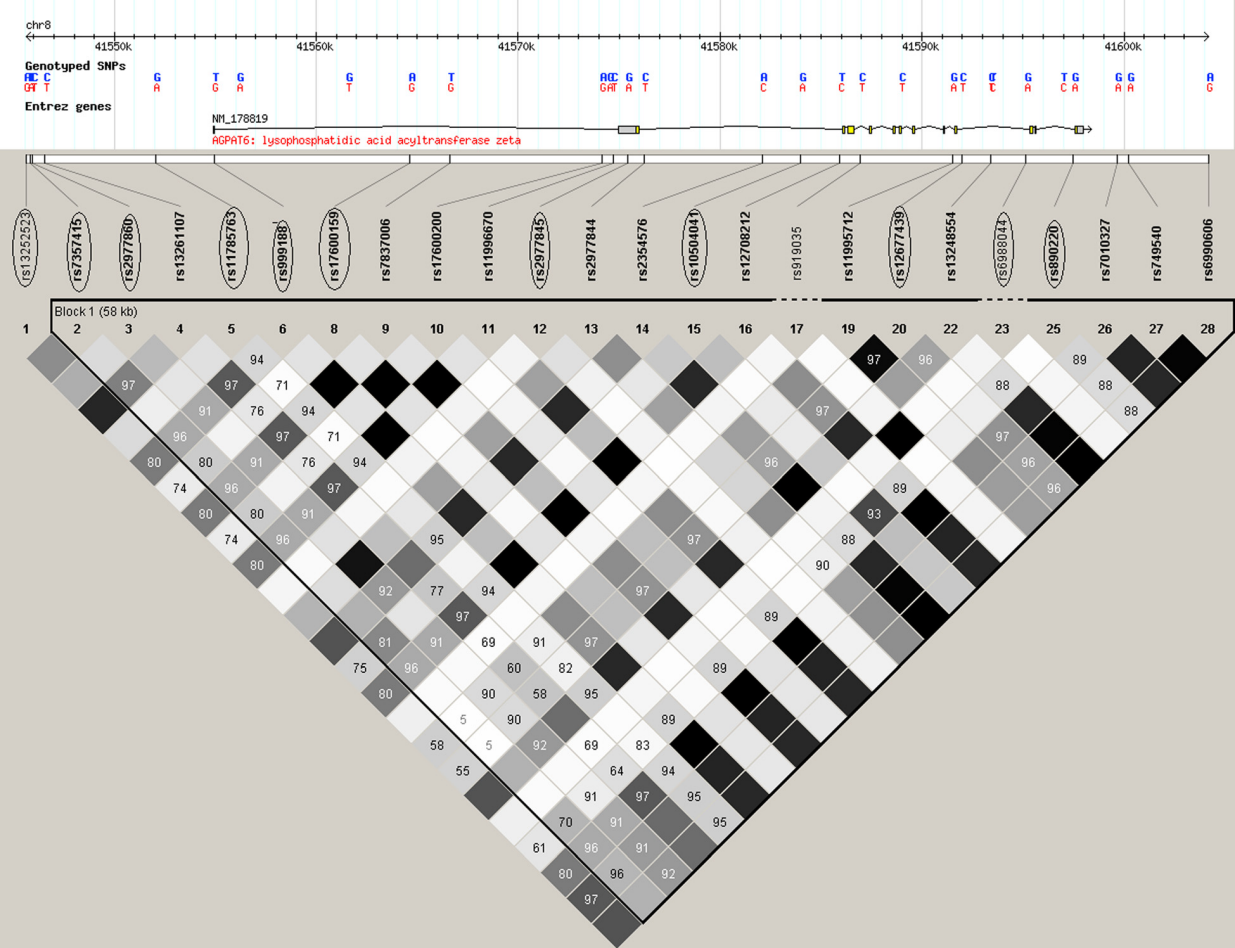
**Linkage disequilibrium (LD) plot across *AGPAT6*.** The box at the top shows the *AGPAT6* gene with the location of the 28 SNPs included in this study. The 11 tag SNPs are indicated by circles around the rs number of the SNPs. The LD plot is based on the measure of D’. Each diamond indicates the pair wise magnitude of LD, with dark grey diamonds indicating strong LD (D’ > 0.8) and light grey: uninformative. LD: linkage disequilibrium is the non-random association of alleles at two or more loci, not necessarily on the same chromosome. Linkage disequilibrium describes a situation in which some combinations of alleles or genetic markers occur more or less frequently in a population than would be expected from a random formation of haplotypes from alleles based on their frequencies.

*SNP selection* We performed tagging of the genomic region including *AGPAT6* (HapMap release 27, Phase II + III, Feb. 09, B36, CEU panel; Chr8:41,544 kb - 41,607 kb (*AGPAT6* position ± 10 kb)) using Haploview 4.2 (http://www.broadinstitute.org/haploview). See LD plot of *AGPAT6* in Figure 1. Eleven SNPs captured all common variation with minor allele frequency (MAF) above 5% (n = 10) and low-frequency variation with MAF below 5% but above 1% (n = 1) variation in *AGPAT6* (r^2^ >0.8). The selected SNPs have the following rs numbers: rs13252523, rs7357415, rs2977860, rs11785763, rs999188, rs17600159, rs2977845, rs10504041, rs12677439, rs6988044 and rs890220.

*Biochemical and anthropometrical measures* Body weight and height were measured in light indoor clothing and without shoes. Waist circumference was measured in the standing position midway between the iliac crest and the lower costal margin. Blood samples were drawn after a 12-h overnight fast. Serum insulin levels were measured by using the AutoDELFIA insulin kit (Perkin-Elmer, Wallac, Turku, Finland). Plasma glucose was analyzed by a glucose oxidase method (Granutest; Merck, Darmstadt, Germany). Serum triglyceride and cholesterols were determined by enzymatic colorimetric methods (GPO-PAP and CHOD-PAP, Roche Molecular Biochemicals).

*Derived estimates of insulin response and insulin sensitivity from an OGTT* Indices of oral glucose-stimulated insulin secretion are reported as the insulinogenic index calculated as ([serum insulin_30 min_ – serum insulin_0 min_ (pmol/l)]/[plasma glucose_30 min_ (mmol/l)]), and the BIGTT-acute insulin response (AIR) index. The surrogate measures of insulin sensitivity (S_I_) are reported as the homeostasis model assessment of insulin resistance (HOMA-IR), Matsuda-S_I_, Stumvoll-S_I_, and BIGTT-S_I_, all calculated as described previously [20-23]. The BIGTT indices, which apply information on sex and BMI, combined with plasma glucose and serum insulin during an OGTT, were calculated as reported [21].

*Genotyping* The eleven *AGPAT6* variants were genotyped using KASPar SNP Genotyping system (KBioscience, Hoddesdon, UK). Success-rates of the genotyping were above 95% for all 11 SNPs. Error rates were below 0.5% for the 11 variants, as estimated from re-genotyping of 596 duplicate samples. The distribution of genotypes was in Hardy-Weinberg equilibrium in the population-based Inter99 for all SNPs (p > 0.05).

*Statistical power* The statistical power in type 2 diabetes case–control studies were calculated using the CaTS power calculator for large genetic association studies (available at http://www.sph.umich.edu/csg/abecasis/cats/). The power calculations assumed a Bonferroni adjusted alpha-value of 0.05/11 = 0.0045, a disease prevalence of 8% and MAFs 5-40% (Additional file 1: Figure S1). Given the MAFs for the 11 investigated *AGPAT6* variants (4.0% - 46%), our study has statistical power of more than 80% to detect an odds ratio (OR) of 1.10 in a case–control design for the most common variants (MAF ~ 40%), whereas we have ~80% power to detect an OR of 1.20-1.25 for the lower frequency variants (MAF ~ 5%). The statistical power calculations for quantitative traits were performed as previously described [15] and showed that we have statistical power of 94% to detect an effect equal to a fraction of a standard deviation of 0.08 for a SNP with a MAF of 46%, while we had power 93% to detect an effect equal to a fraction of a standard deviation of 0.20 in case of a MAF 5%. Our power calculations do not allow any solid conclusions. Additional file 1: Figure S1 is a graph showing power for a range of OR’s and allele frequencies.

*Statistics* A case–control analysis of type 2 diabetes was performed using logistic regression to examine differences in genotypes assuming an additive genetic model with adjustment for sex and age. A general linear model was used to test quantitative variables for differences between genotype groups applying an additive model adjusted for age and sex. We also performed the analysis including BMI as covariate, however that did not add any information to the analysis and results are not shown. Values of serum insulin, Matsuda-S_I_ and Stumvoll-S_I_ were logarithmically transformed before analyses, since that was appropriate when checked for normality of the residuals. A p-value below a Bonferroni corrected threshold of 0.05/11 = 0.0045 was considered to be significant, taking the 11 variants into account. All analyses were performed in RGui version 2.11.1 (available at http://www.r-project.org).

## Results

Eleven common and low-frequency *AGPAT6* variants were genotyped in 12,068 Danish individuals All the 11 genotyped *AGPAT6* variants were in low pairwise linkage disequilibrium (r^2^ < 0.46).

In case–control settings, four of the variants, rs2977845, rs10504041, rs6988044 and rs890220 were nominally associated with type 2 diabetes, when examined in 4,638 type 2 diabetes and 5,934 control individuals (p < 0.05) (Table 1). For all the variants we calculated OR for the minor allele in a model adjusted for age and sex. Thus, carriers of the minor allele of rs2977845 (OR1.18 [95% CI 1.01-1.37, p = 0.034]) had a nominally increased risk of type 2 diabetes, while carriers of the minor alleles of rs10504041 (OR 0.91 [95% CI 0.84-0.99, p = 0.030]), rs6988044 (OR 0.87 [95% CI 0.76-0.99, p = 0.030]) and rs890220 (OR 0.88 [95% CI 0.8-0.96, p = 0.0052]) showed a nominally reduced susceptibility to type 2 diabetes. However, after correction for multiple testing none of the 11 variants were robustly associated with type 2 diabetes (Table 1). We performed lookup of the genotyped SNPs in results from the newest DIAGRAM genome-wide association study (http://diagram-consortium.org/downloads.html) [24]. Seven of the 11 SNPs were found in DIAGRAM results. For the correlated rs10504041 and rs890220 SNPs nominal association in DIAGRAM data was observed yet in the opposite direction compared to the present data (Additional file 1: Table S11). The minor G allele of rs17600159 showed nominal association in DIAGRAM data (OR 1.10, *P* = 0.0041) but was not statistical significant in the current data (OR 1.12, *P* = 0.09). (Additional file 1: Table S2).

**Table 1:** **Type 2 diabetes association for the 11 *AGPAT6 *** **variants genotyped in type 2 diabetic individuals and control individuals with normal glucose tolerance**

***AGPAT6 *** **variant**	**N**		**Minor:major allele**	**MAF**	**Odds ratio**	**P-value**
					**(95% CI)**	
	**T2D**	**NGT**				
	**(wildtype/hetero-/homozygous)**	**(wildtype/hetero-/homozygous)**				
rs13252523	1428/2304/802	1821/2840/1148	G:A	0.44	0.95 (0.88-1.01)	0.098
rs7357415	2779/1555/197	3546/1973/293	T:A	0.22	0.95 (0.87-1.03)	0.19
rs2977860	2158/1931/386	2827/2419/478	T:C	0.30	1.03 (0.95-1.1)	0.49
rs11785763	3364/1095/94	4307/1405/116	G:A	0.14	0.99 (0.90-1.08)	0.79
rs999188	1379/2271/875	1802/2817/1169	T:G	0.44	1.00 (0.94-1.07)	0.98
rs17600159	3941/581/13	5123/658/16	G:A	0.063	1.12 (0.98-1.29)	0.092
rs2977845	4124/433/12	5332/513/15	G:A	0.048	1.18 (1.01-1.37)	0.034
rs10504041	2980/1359/169	3738/1814/256	G:A	0.19	0.91 (0.84-0.99)	0.030
rs12677439	2568/1709/271	3374/2062/343	T:C	0.24	1.06 (0.99-1.15)	0.11
rs6988044	3993/566/15	5028/786/31	G:A	0.069	0.87 (0.76-0.99)	0.030
rs890220	3376/1107/85	4188/1468/151	G:A	0.15	0.88 (0.80-0.96)	0.0052

P-values and odds ratios were calculated by logistic regression analysis assuming an additive genetic model and adjusting for age and sex. Effects were calculated for the minor allele. *T2D* Type 2 diabetes, *NGT* Normal glucose tolerance, *MAF* Minor allele frequency.

We investigated the relationships of the 11 *AGPAT6* variants with quantitative metabolic traits in the population-based Inter99 cohort of 5,645 non-diabetic Danes. Based on its function as GPAT4 and lessons learned from *agpat6*-deficient mice, we focused on measures of obesity (BMI and waist), dyslipidemia (triglycerides, HDL-cholesterol and total-cholesterol), fasting and 2-h post-glucose load levels of insulin or glucose, and OGTT-derived indices of insulin sensitivity (HOMA-IR, BIGTT-S_I_, Stumvoll-S_I_, Matsuda S_I_) and insulin secretion (Insulinogenic index and BIGTT-AIR). All variants were analysed applying an additive genetic model adjusting for age and sex. The minor G-allele of rs17600159 showed nominal association with plasma glucose and insulin 2-h after an OGTT (Table 2). However, this was not significant after correction for multiple testing. For the minor A-allele of rs890220, which showed the most significant nominal association with type 2 diabetes, we observed no association with any of the examined metabolic traits (Table 3). Moreover, we could not demonstrate any association of the other nine *AGPAT6* variants with these traits of obesity, dyslipidemia, insulin sensitivity or insulin secretion (Additional file 1: Tables S3–11). Adjustment for BMI in all analysis did not change the results substantially.

**Table 2:** **Anthropometric and quantitative metabolic characteristics of 5,645 middle-aged Danish Inter99 participants stratified according to the *AGPAT6 *** **rs17600159 genotype**

***AGPAT6 *** **rs17600159**	**AA**	**GA**	**GG**	**p value**
N (men/women)	4835(2373/2462)	641(317/324)	19(10/9)	
Age (years)	45.9 ± 7.9	45.8 ± 7.5	46.7 ± 7.9	
Waist (cm)	85.9 ± 12.9	85.9 ± 12.9	90.7 ± 12.9	0.56
BMI (kg/m^2^)	26.0 ± 4.4	25.9 ± 4.4	27.8 ± 5.1	0.82
Serum Triglycerides (mmol/l)	1.3 ± 1.4	1.3 ± 1.1	1.2 ± 0.5	0.34
Serum Total cholesterol (mmol/l)	5.5 ± 1.1	5.5 ± 1.0	5.2 ± 1.0	0.92
Serum HDL cholesterol (mmol/l)	1.4 ± 0.4	1.4 ± 0.4	1.4 ± 0.3	0.61
**Plasma glucose** (mmol/l)				
Fasting	5.45 ± 0.51	5.47 ± 0.51	5.43 ± 0.55	0.36
120 min	5.93 ± 1.52	6.06 ± 1.64	6.76 ± 1.57	0.025
**Serum insulin** (pmol/l)				
Fasting	41 ± 26	40.3 ± 24.4	41.8 ± 22.7	0.85
120 min	206 ± 198	212 ± 184	374 ± 410	0.013
**Insulin secretion indices**				
Insulinogenic index	29.8 ± 19.7	30.2 ± 18.8	35.0 ± 30.4	0.32
BIGTT-AIR	1860 ± 1096	1859 ± 1017	2372 ± 2177	0.59
**Insulin sensitivity indices**				
HOMA-IR (pmol x mmol/l)	9.98 ± 6.84	9.95 ± 6.42	10.09 ± 5.44	0.80
BIGTT-S_I_	9.49 ± 3.94	9.26 ± 3.94	7.97 ± 4.42	0.075
Stumvoll S_I_	0.10 ± 0.02	0.10 ± 0.02	0.09 ± 0.03	0.34
Matsuda S_I_	9.19 ± 5.69	8.94 ± 5.69	7.80 ± 4.56	0.15

Data are mean ± standard deviation. Values of serum insulin, the Stumvoll-S_I_ and the Matsuda-S_I_ were logarithmically transformed before analyses. P-values were calculated assuming an additive genetic model with adjustment for age and sex.

**Table 3:** **Anthropometric and quantitative metabolic characteristics of 5,645 middle-aged Danish Inter99 participants stratified according to the *AGPAT6 *** **rs890220 genotype**

***AGPAT6 *** **rs890220**	**AA**	**GA**	**GG**	**p value**
N (men/women)	3995(1976/2019)	1368(654/714)	134(67/67)	
Age (years)	45.8 ± 7.9	46.3 ± 7.7	45.9 ± 8.0	
Waist (cm)	85.9 ± 13.0	86.1 ± 12.8	83.3 ± 12.1	0.48
BMI (kg/m^2^)	26.0 ± 4.5	26.1 ± 4.2	25.4 ± 4.0	0.60
Serum Triglycerides (mmol/l)	1.3 ± 1.5	1.3 ± 0.9	1.3 ± 1.2	0.50
Serum Total cholesterol (mmol/l)	5.5/-1.1	5.6 ± 1.0	5.5 ± 1.1	0.084
Serum HDL cholesterol (mmol/l)	1.4 ± 0.4	1.5 ± 0.4	1.4 ± 0.4	0.86
**Plasma glucose** (mmol/l)				
Fasting	5.45 ± 0.52	5.47 ± 0.50	5.42 ± 0.50	0.74
120 min	5.95 ± 1.56	5.96 ± 1.51	5.76 ± 1.50	0.44
**Serum insulin** (pmol/l)				
Fasting	41 ± 27	40 ± 25	39 ± 23	0.74
120 min	209 ± 199	201 ± 189	193 ± 146	0.63
**Insulin secretion indices**				
Insulinogenic index	29.7 ± 19.3	30.0 ± 20.1	33.0 ± 23.1	0.22
BIGTT-AIR	1861 ± 1088	1852 ± 1067	1930 ± 1210	0.63
**Insulin sensitivity indices**				
HOMA-IR (pmol x mmol/l)	10.03 ± 6.85	9.96 ± 6.796	9.58 ± 6.21	0.81
BIGTT-S_I_	9.44 ± 4.00	9.42 ± 3.79	9.54 ± 3.73	0.26
Stumvoll S_I_	0.10 ± 0.02	0.10 ± 0.02	0.10 ± 0.02	0.085
Matsuda S_I_	9.17 ± 5.78	9.04 ± 5.34	9.20 ± 6.47	0.85

Data are mean ± standard deviation. Values of serum insulin, the Stumvoll-S_I_ and the Matsuda-S_I_ were logarithmically transformed before analyses. P-values were calculated assuming an additive genetic model with adjustment for age and sex.

## Discussion

Several studies have shown that ectopic deposition of triglycerides in lipid droplets is strongly associated with insulin resistance and plays an important role in the pathogenesis of type 2 diabetes [6-8,25]. This suggests that variation in genes related to *de novo* triglyceride synthesis and lipid droplet formation could be associated with type 2 diabetes or related metabolic traits. Here, we have tested whether common or low-frequency variation in *AGPAT6*, was associated with increased susceptibility of type 2 diabetes or related metabolic traits. In contrast to our hypothesis, we could not demonstrate a robust association with type 2 diabetes for any of the eleven *AGPAT6* variants investigated. Moreover, none of these variants were significantly associated with measures of adiposity, dyslipidemia or indices of insulin sensitivity or insulin secretion in a large cohort of non-diabetic, middle-aged Danish individuals. These results indicate that common and low-frequency variation in the gene encoding the most recently identified glycerol-3-phosphate acyltransferase, GPAT4, does not play a major role in the presence or absence of prediabetic phenotypes or risk of type 2 diabetes.

In previous genome-wide association (GWA) studies of genetic variants contributing to type 2 diabetes risk [26-29], the majority of genes investigated included only variants with a MAF about 5% or higher. In none of these studies, variants in genes regulating *de novo* phospholipid or triglyceride synthesis or controlling lipid droplet formation have been found to be robustly associated with type 2 diabetes [30]. Here, we applied the candidate gene approach to capture not only common but also low-frequency variation in *AGPAT*6 to examine its potential association with type 2 diabetes. However, in agreement with the GWA-studies, we were unable to show any robust association of genetic variation in *AGPAT6* with type 2 diabetes in a Danish population. Yet, the statistical power of the current study does not allow for solid conclusions on the possible existence of common *AGPAT6* variants with modest or subtle effect on disease risk. Also, this finding does not exclude that rare *AGPAT6* variants with a MAF less than 1% may cause type 2 diabetes associated with e.g. lipodystrophy.

Studies of humans with acquired and genetic syndromes of lipodystrophy have clearly shown that failure of lipid storage in adipose tissues, hypertriglyceridemia and ectopic deposition of lipid in liver and skeletal muscle is associated with severe insulin resistance [9]. Mutations in *AGPAT2*, which is another gene involved in *de novo* triglyceride synthesis, is an accepted cause of congenital generalized lipodystrophy associated with extreme insulin resistance and early onset diabetes in humans [31], and the phenotype has been reproduced in *agpat2*-deficient mice [32]. Moreover, accumulation of triglycerides in skeletal muscle is negatively associated with insulin sensitivity even in a healthy population [2-5]. These findings, together with reports of lower weight, resistance to obesity and depletion of triglyceride in adipose tissues in *agpat6*-deficient mice, suggested a possible role for variation in *AGPAT6* in relation to prediabetic phenotypes such as obesity, circulating levels of triglycerides and insulin resistance. However, at least in a non-diabetic population of Danish individuals, we were unable to demonstrate any significant association of common and low-frequency variation in *AGPAT6* with these diabetes-related traits. These results are consistent with the absence of *AGPAT6* variants among genetic variants shown to contribute to dyslipidemia and hypertriglyceridemia in current available GWA-studies [33].

The study has following limitations. First, the case–control analysis is limited by sample size. We cannot exclude association in our samples with odds ratios outside of the 95% confidential intervals, i.e. for rs13252523, we cannot exclude an association with type 2 diabetes with on odds ratio higher than 1.01 or lower than 0.88. Secondly, although we corrected for multiple testing for the 11 variants, we did not correct for the number of phenotypes since they are not independent, and therefore would give an over-correction by Bonferroni. Another limitation of the present study is that association of *AGPAT*6 variants with insulin secretion and insulin sensitivity was based on OGTT-derived surrogate measures of these traits rather than application of gold-standard methods. A recent report indicates that, in particular, caution is required for the interpretation of differences in OGTT-derived values of insulin sensitivity, because such differences may reflect variations in beta-cell function rather than true variations in insulin sensitivity [34]. It would also have been interesting to examine association of these *AGPAT6* variants with direct or indirect measures of ectopic lipid content in relevant target tissues such as liver and skeletal muscle, and with *AGPAT6* expression and/or activity in these tissues. Nevertheless, so far there is no evidence from GWA studies that common variants in *AGPAT6* associate with hepatic steatosis such as seen in non-alcoholic fatty liver disease [35,36].

## Conclusions

In summary, we here report that common and low-frequency variation in *AGPAT6* do not associate with type 2 diabetes, or related obvious metabolic phenotypes such as obesity, dyslipidemia or insulin resistance in a Danish population. Further studies are warranted to exclude a role for other genes involved in *de novo* triglyceride synthesis and lipid droplet formation.

## Abbreviations

AIR: Acute insulin response; BAT: Brown adipose tissue; GPAT4: Glycerol-3-phosphate acyltransferase 4; HOMA: Homeostasis model assessment; LDs: Lipid droplets; OGTT: Oral glucose tolerance test; WAT: White adipose tissue.

## Competing interests

D. R. Witte was employed by Steno Diabetes Center which is a teaching hospital facility working in the Danish National Health Service and owned by Novo Nordisk.

## Authors’ contributions

LSS collected and analysed the data and wrote the manuscript. NG, KB, MW analyzed the data and reviewed/edited the manuscript. TJ, DW, TL, AAN, IB, CC collected the patient data and reviewed/edited manuscript. OP, KY analysed the data and reviewed/edited manuscript. KH designed the study and wrote the manuscript. TH, JEH and HBN designed the study and reviewed/edited manuscript. All authors read and approved the final manuscript.

## Pre-publication history

The pre-publication history for this paper can be accessed here:

http://www.biomedcentral.com/1471-2350/14/113/prepub

## Supplementary Material

Additional file 1: Figure S1 Graph for power for a range of lower ORs and allele frequencies**.** Table S1 Clinical characteristics of the four study groups. Data are means ± standard deviation. ∗, in the ADDITION study fasting glucose is measured on capillary blood. T2D, type 2 diabetes. **Table S2** Data on 7 of the SNPs from the newest DIAGRAM database. OR’s are mean ± 95% CI. T2D, type 2 diabetes; NGT, normal glucose tolerance; MAF, minor allele frequency. **Table S3-S11** Anthropometric and metabolic characteristics of 5,645 middle-aged Danish Inter99 participants stratified according to *AGPAT6* rs999188 **(Table S2)**, 12677439 **(Table S3)**, rs6988044 **(Table S4)**, rs2977860 **(Table S5)**, rs13252523 **(Table S6)**, rs2977845 **(Table S7)**, rs11785763 **(Table S8)**, rs7357415 **(Table S9)**, and rs10504041 **(Table S10)** genotypes. Data are mean ± standard deviation. Values of serum insulin, the Stumvoll-S_I_ and the Matsuda-S_I_ were logarithmically transformed before analyses. P-values were calculated assuming an additive genetic model with adjustment for age and sex. Click here for file
