# Supplementary material for: Studies of association of AGPAT6 variants with type 2 diabetes and related metabolic phenotypes in 12,068 Danes
Source: BMC Med Genet. 2013 Oct 25;14:113. doi: 10.1186/1471-2350-14-113 (PMC4231429; doi:10.1186/1471-2350-14-113)
Supplement: Additional file 1: Figure S1 — Graph for power for a range of lower ORs and allele frequencies. Table S1 Clinical characteristics of the four study groups. Data are means ± standard deviation. ∗, in the ADDITION study fasting glucose is measured on capillary blood. T2D, type 2 diabetes. Table S2 Data on 7 of the SNPs from the newest DIAGRAM database. OR’s are mean ± 95% CI. T2D, type 2 diabetes; NGT, normal glucose tolerance; MAF, minor allele frequency. Table S3-S11 Anthropometric and metabolic characteristics of 5,645 middle-aged Danish Inter99 participants stratified according to AGPAT6 rs999188 (Table S2), 12677439 (Table S3), rs6988044 (Table S4), rs2977860 (Table S5), rs13252523 (Table S6), rs2977845 (Table S7), rs11785763 (Table S8), rs7357415 (Table S9), and rs10504041 (Table S10) genotypes. Data are mean ± standard deviation. Values of serum insulin, the Stumvoll-SI and the Matsuda-SI were logarithmically transformed before analyses. P-values were calculated assuming an additive genetic model with adjustment for age and sex. [file 1471-2350-14-113-S1.docx]

**Additional file**

**Figure S1** Graph for power for a range of lower ORs and allele frequencies.

**Table S1** Clinical characteristics of the four study groups. Data are means ± standard deviation. ∗, in the ADDITION study fasting glucose is measured on capillary blood. T2D, type 2 diabetes.

**Table S2** Data on 7 of the SNPs from the newest DIAGRAM database. OR’s are mean ± 95% CI. T2D, type 2 diabetes; NGT, normal glucose tolerance; MAF, minor allele frequency.

**Table S3-S11** Anthropometric and metabolic characteristics of 5,645 middle-aged Danish Inter99 participants stratified according to *AGPAT6* rs999188 (Table S2), 12677439 (Table S3), rs6988044 (Table S4), rs2977860 (Table S5), rs13252523 (Table S6), rs2977845 (Table S7), rs11785763 (Table S8), rs7357415 (Table S9), and rs10504041 (Table S10) genotypes. Data are mean ± standard deviation. Values of serum insulin, the Stumvoll-S_I_ and the Matsuda-S_I_ were logarithmically transformed before analyses. P-values were calculated assuming an additive genetic model with adjustment for age and sex.

**Figure S1**

**Table S1** Overview of the clinical characteristics of the four study groups.

|  | **Inter99** | **Steno** | | **ADDITION** | **Vejle Biobank** | |
| --- | --- | --- | --- | --- | --- | --- |
|  |  | **T2D Cases** | **Controls** |  | **T2D cases** | **Controls** |
| ***N*** | 6,287 | 1,658 | 567 | 1,575 | 1030 | 951 |
| **Men/women** | 3,073/3,214 | 1,046/612 | 290/277 | 898/677 | 615/415 | 368/583 |
| **Age (years)** | 46.2 ± 7.9 | 62.1 ± 11.3 | 56.6 ± 10.0 | 60.0 ± 6.8 | 63.1 ± 8.6 | 58.2 ± 12.1 |
| **BMI (kg/m2)** | 26.3 ± 4.6 | 30.1 ± 5.6 | 25.8 ± 3,7 | 28.6 ± 4.9 | 30.5 ± 5.6 | 24.5 ± 3.9 |
| **HbA1c (%)** | 5.9 ± 0.6 | 8.1 ± 1.6 | 5.8 ± 0.5 | 5.9 ± 0.7 | 7.1 ± 1.1 | 5.4 ± 0.3 |
| **Fasting glucose (mmol/l)** | 5.6 ± 1.1 | 9.7 ± 3.5 | 5.1 ± 0.4 | 5.4 ± 1.2 * | 8.7 ± 2.6 | 5.1 ± 0.3 |

**Table S2** Type 2 diabetes association for 7 *AGPAT6* variants available in the newest DIAGRAM dataset (24).

| *AGPAT6* variant | Risk:Other  allele | P-value | Odds Ratio (95% CI) | N | |
| --- | --- | --- | --- | --- | --- |
|  |  |  |  | T2D | NGT |
| rs13252523 | G:A | 0.61 | 1.01 (0.98-1.04) | 12171 | 56862 |
| rs7357415 | A:T | 0.31 | 1.02 (0.98-1.06) | 12171 | 56862 |
| rs2977860 | C:T | 0.7 | 1.01(0.97-1.05) | 9580 | 53810 |
| rs11785763 | G:A | 0.78 | 1.01 (0.96-1.06) | 9580 | 53810 |
| rs17600159 | G:A | 0.0041 | 1.1 (1.03-1.18) | 12171 | 56862 |
| rs10504041 | G:A | 0.06 | 1.04 (1.00-1.09) | 12171 | 56862 |
| rs6988044 | G:A | 0.029 | 1.08 (1.01-1.17) | 8558 | 52735 |

(<http://diagram-consortium.org/downloads.html>) (24)

**Table S3** Anthropometric and quantitative metabolic characteristics of 5,645 middle-aged Danish Inter99 participants stratified according to the *AGPAT6* rs999188 genotype

| *AGPAT6* rs999188 | AA | GA | GG | p value |
| --- | --- | --- | --- | --- |
| N (men/women) | 1723(852/871) | 2681(1328/1353) | 1100(527/573) |  |
| Age (years) | 45.9±7.9 | 45.9±7.9 | 46.1±7.8 |  |
| Waist (cm) | 86.1±13.2 | 85.9±12.7 | 85.7±12.6 | 0.61 |
| BMI (kg/m^2^) | 26.1±4.5 | 26.0±4.3 | 26.0±4.4 | 0.71 |
| Serum Triglycerides (mmol/l) | 1.3±1.3 | 1.3±1.5 | 1.3±1.0 | 0.40 |
| Serum Total cholesterol (mmol/l) | 5.5±1.1 | 5.5±1.1 | 5.5±1.0 | 0.22 |
| Serum HDL cholesterol (mmol/l) | 1.4±0.4 | 1.4±0.4 | 1.4±0.4 | 0.89 |
| **Plasma glucose** (mmol/l) |  |  |  |  |
| Fasting | 5.45±0.51 | 5.45±0.51 | 5.46±0.50 | 0.43 |
| 120 min | 5.94±1.56 | 5.95±1.53 | 6.0±1.56 | 0.70 |
| **Serum insulin** (pmol/l) |  |  |  |  |
| Fasting | 41±27 | 40±26 | 41±27 | 0.49 |
| 120 min | 208±206 | 205±188 | 206±200 | 0.82 |
| **Insulin secretion indices** |  |  |  |  |
| Insulinogenic index | 29.8±19.4 | 29.8±19.0 | 29.9±21.3 | 0.81 |
| BIGTT-AIR | 1864±1124 | 1855±1043 | 1870±1160 | 0.85 |
| **Insulin sensitivity indices** |  |  |  |  |
| HOMA-IR (pmol*mmol/l) | 9.95±6.90 | 9.95±6.63 | 10.19±7.17 | 0.43 |
| BIGTT-S_I_ | 9.49±4.03 | 9.45±3.89 | 9.43±3.97 | 0.98 |
| Stumvoll S_I_ | 0.10±0.02 | 0.10±0.02 | 0.10±0.02 | 0.63 |
| Matsuda S_I_ | 9.24±5.54 | 9.05±5.54 | 9.23±6.14 | 0.59 |

**Table S4** Anthropometric and quantitative metabolic characteristics of 5,645 middle-aged Danish Inter99 participants stratified according to the *AGPAT6* rs12677439 genotype

| *AGPAT6* rs12677439 | AA | GA | GG | p value |
| --- | --- | --- | --- | --- |
| N (men/women) | 3197(1569/1628) | 1963(971/992) | 326(151/175) |  |
| Age (years) | 46.0±7.8 | 45.9±8.0 | 45.8±7.6 |  |
| Waist (cm) | 86.0±12.9 | 85.9±12.7 | 85.5±12.8 | 0.89 |
| BMI (kg/m^2^) | 26.0±4.3 | 26.0±4.4 | 26.0±4.4 | 0.79 |
| Serum Triglycerides (mmol/l) | 1.3±1.2 | 1.3±1.7 | 1.3±0.8 | 0.2 |
| Serum Total cholesterol (mmol/l) | 5.5±1.1 | 5.5±1.0 | 5.5±1.0 | 0.47 |
| Serum HDL cholesterol (mmol/l) | 1.4±0.4 | 1.4±0.4 | 1.4±0.4 | 0.59 |
| **Plasma glucose** (mmol/l) |  |  |  |  |
| Fasting | 5.45±0.51 | 5.46±0.51 | 5.46±0.50 | 0.28 |
| 120 min | 5.92±1.52 | 6.00±1.58 | 5.94±1.55 | 0.23 |
| **Serum insulin** (pmol/l) |  |  |  |  |
| Fasting | 40±26 | 41±27 | 40±28 | 0.63 |
| 120 min | 203±193 | 212±198 | 205±188 | 0.23 |
| **Insulin secretion indices** |  |  |  |  |
| Insulinogenic index | 29.9±19.2 | 29.8±20.3 | 29.0±19.4 | 0.31 |
| BIGTT-AIR | 1856±1059 | 1874±1138 | 1820±1010 | 0.52 |
| **Insulin sensitivity indices** |  |  |  |  |
| HOMA-IR (pmol*mmol/l) | 9.88±6.75 | 10.19±6.88 | 9.97±7.39 | 0.54 |
| BIGTT-S_I_ | 9.49±3.89 | 9.35±4.01 | 9.51±4.05 | 0.21 |
| Stumvoll S_I_ | 0.10±0.02 | 0.10±0.02 | 0.10±0.02 | 0.2 |
| Matsuda S_I_ | 9.15±5.33 | 9.07±6.13 | 9.42±5.89 | 0.35 |

**Table S5** Anthropometric and quantitative metabolic characteristics of 5,645 middle-aged Danish Inter99 participants stratified according to the *AGPAT6* rs6988044 genotype

| *AGPAT6* rs6988044 | AA | GA | GG | p value |
| --- | --- | --- | --- | --- |
| N (men/women) | 4803(2337/2466) | 724(380/344) | 27(14/13) |  |
| Age (years) | 45.9±7.9 | 46.5±7.7 | 46.9±8.6 |  |
| Waist (cm) | 85.9±13.0 | 86.5±12.2 | 83.9±12.6 | 0.73 |
| BMI (kg/m^2^) | 26.0±4.4 | 26.2±4.5 | 25.0±4.2 | 0.81 |
| Serum Triglycerides (mmol/l) | 1.3±1.4 | 1.3±1.2 | 1.3±0.9 | 0.67 |
| Serum Total cholesterol (mmol/l) | 5.5±1.1 | 5.5±1.0 | 5.5±1.0 | 0.95 |
| Serum HDL cholesterol (mmol/l) | 1.4±0.4 | 1.4±0.4 | 1.4±0.3 | 0.29 |
| **Plasma glucose** (mmol/l) |  |  |  |  |
| Fasting | 5.45±0.51 | 5.49±0.50 | 5.39±0.48 | 0.37 |
| 120 min | 5.96±1.54 | 5.93±1.51 | 6.26±1.76 | 0.84 |
| **Serum insulin** (pmol/l) |  |  |  |  |
| Fasting | 41±26 | 41±26 | 37±23 | 0.86 |
| 120 min | 207±195 | 207±209 | 250±258 | 0.88 |
| **Insulin secretion indices** |  |  |  |  |
| Insulinogenic index | 29.7±19.4 | 30.3±20.0 | 34.9±27.2 | 0.34 |
| BIGTT-AIR | 1855±1049 | 1860±1076 | 2310±2386 | 0.59 |
| **Insulin sensitivity indices** |  |  |  |  |
| HOMA-IR (pmol*mmol/l) | 9.99±6.85 | 10.11±6.64 | 8.92±6.00 | 0.77 |
| BIGTT-S_I_ | 9.46±3.96 | 9.31±3.85 | 10.07±4.43 | 0.66 |
| Stumvoll S_I_ | 0.10±0.02 | 0.10±0.02 | 0.10±0.02 | 0.78 |
| Matsuda S_I_ | 9.18±5.76 | 8.92±5.24 | 9.35±4.72 | 0.61 |

**Table S6** Anthropometric and quantitative metabolic characteristics of 5,645 middle-aged Danish Inter99 participants stratified according to the *AGPAT6* rs2977860 genotype

| *AGPAT6* rs2977860 | AA | GA | GG | p value |
| --- | --- | --- | --- | --- |
| N (men/women) | 2683(1299/1384) | 2280(1136/1144) | 485(241/244) |  |
| Age (years) | 46.1±7.8 | 45.8±7.9 | 45.7±7.8 |  |
| Waist (cm) | 85.8±12.7 | 85.9±13.0 | 86.3±12.7 | 0.63 |
| BMI (kg/m^2^) | 26.0±4.3 | 26.0±4.3 | 26.1±4.6 | 0.99 |
| Serum Triglycerides (mmol/l) | 1.3±1.0 | 1.3±1.7 | 1.2±0.7 | 0.76 |
| Serum Total cholesterol (mmol/l) | 5.5±1.1 | 5.5±1.1 | 5.5±1.1 | 0.66 |
| Serum HDL cholesterol (mmol/l) | 1.4±0.4 | 1.4±0.4 | 1.4±0.4 | 0.72 |
| **Plasma glucose** (mmol/l) |  |  |  |  |
| Fasting | 5.46±0.52 | 5.44±0.51 | 5.46±0.49 | 0.44 |
| 120 min | 5.96±1.52 | 5.93±1.55 | 6.04±1.59 | 0.58 |
| **Serum insulin** (pmol/l) |  |  |  |  |
| Fasting | 41±26 | 40±26 | 40±27 | 0.19 |
| 120 min | 206±192 | 205±192 | 212±225 | 0.66 |
| **Insulin secretion indices** |  |  |  |  |
| Insulinogenic index | 30.1±20.6 | 29.5±18.6 | 29.2±17.9 | 0.37 |
| BIGTT-AIR | 1860±1064 | 1866±1131 | 1823±869 | 0.94 |
| **Insulin sensitivity indices** |  |  |  |  |
| HOMA-IR (pmol*mmol/l) | 10.10±6.83 | 9.87±6.76 | 9.89±7.01 | 0.19 |
| BIGTT-S_I_ | 9.41±3.89 | 9.52±3.98 | 9.4±3.95 | 0.93 |
| Stumvoll S_I_ | 0.10±0.02 | 0.10±0.02 | 0.10±0.02 | 0.57 |
| Matsuda S_I_ | 9.04±5.60 | 9.24±5.73 | 9.40±5.91 | 0.16 |

**Table S7** Anthropometric and quantitative metabolic characteristics of 5,645 middle-aged Danish Inter99 participants stratified according to the *AGPAT6* rs13252523 genotype

| *AGPAT6* rs13252523 | AA | GA | GG | p value |
| --- | --- | --- | --- | --- |
| N (men/women) | 1765(855/910) | 2670(1338/1332) | 1077(515/562) |  |
| Age (years) | 45.9±8.0 | 45.9±7.8 | 46.2±7.9 |  |
| Waist (cm) | 86.2±13.2 | 85.9±12.8 | 85.5±12.4 | 0.098 |
| BMI (kg/m^2^) | 26.2±4.6 | 26.0±4.3 | 25.9±4.2 | 0.12 |
| Serum Triglycerides (mmol/l) | 1.3±1.2 | 1.3±1.5 | 1.3±1.0 | 0.69 |
| Serum Total cholesterol (mmol/l) | 5.5±1.1 | 5.5±1.1 | 5.5±1.0 | 0.74 |
| Serum HDL cholesterol (mmol/l) | 1.4±0.4 | 1.4±0.4 | 1.4±0.4 | 0.89 |
| **Plasma glucose** (mmol/l) |  |  |  |  |
| Fasting | 5.44±0.51 | 5.46±0.52 | 5.46±0.50 | 0.22 |
| 120 min | 5.95±1.56 | 5.9±1.52 | 5.97±1.56 | 0.86 |
| **Serum insulin** (pmol/l) |  |  |  |  |
| Fasting | 40±26 | 40±26 | 42±28 | 0.33 |
| 120 min | 208±202 | 205±192 | 210±201 | 0.47 |
| **Insulin secretion indices** |  |  |  |  |
| Insulinogenic index | 29.4767±18.7596 | 29.8813±19.3882 | 30.2773±21.3423 | 0.36 |
| BIGTT-AIR | 1849±939 | 1871±1172 | 1854±1078 | 0.69 |
| **Insulin sensitivity indices** |  |  |  |  |
| HOMA-IR (pmol*mmol/l) | 9.93±6.86 | 9.95±6.70 | 10.26±7.09 | 0.26 |
| BIGTT-S_I_ | 9.50±4.05 | 9.41±3.86 | 9.43±3.99 | 0.88 |
| Stumvoll S_I_ | 0.10±0.02 | 0.10±0.02 | 0.10±0.02 | 0.83 |
| Matsuda S_I_ | 9.25±5.55 | 9.09±5.72 | 9.07±5.80 | 0.28 |

**Table S8** Anthropometric and quantitative metabolic characteristics of 5,645 middle-aged Danish Inter99 participants stratified according to the *AGPAT6* rs2977845 genotype

| *AGPAT6* rs2977845 | AA | GA | GG | p value |
| --- | --- | --- | --- | --- |
| N (men/women) | 5038(2476/2562) | 505(246/259) | 19(11/8) |  |
| Age (years) | 45.9±7.8 | 46.0±7.9 | 44.4±6.9 |  |
| Waist (cm) | 85.9±12.8 | 86.1±13.1 | 87.2±10.8 | 0.66 |
| BMI (kg/m^2^) | 26.0±4.4 | 26.1±4.5 | 26.7±4.7 | 0.67 |
| Serum Triglycerides (mmol/l) | 1.3±1.4 | 1.2±0.7 | 1.4±0.8 | 0.98 |
| Serum Total cholesterol (mmol/l) | 5.5±1.1 | 5.6±1.0 | 5.5±0.9 | 0.22 |
| Serum HDL cholesterol (mmol/l) | 1.4±0.4 | 1.5±0.4 | 1.5±0.4 | 0.11 |
| **Plasma glucose** (mmol/l) |  |  |  |  |
| Fasting | 5.46±0.52 | 5.42±0.48 | 5.47±0.43 | 0.27 |
| 120 min | 5.95±1.53 | 5.89±1.57 | 6.70±1.87 | 0.85 |
| **Serum insulin** (pmol/l) |  |  |  |  |
| Fasting | 41±26 | 40±26 | 34±16 | 0.27 |
| 120 min | 207±196 | 208±213 | 180±118 | 0.76 |
| **Insulin secretion indices** |  |  |  |  |
| Insulinogenic index | 30.0±20.0 | 28.4±16.2 | 23.7±18.8 | 0.11 |
| BIGTT-AIR | 1870±1121 | 1799±744 | 1672±743 | 0.54 |
| **Insulin sensitivity indices** |  |  |  |  |
| HOMA-IR (pmol*mmol/l) | 10.04±6.84 | 9.76±6.80 | 8.21±4.06 | 0.22 |
| BIGTT-S_I_ | 9.42±3.94 | 9.67±3.99 | 9.18±3.33 | 0.36 |
| Stumvoll S_I_ | 0.10±0.02 | 0.10±0.02 | 0.10±0.02 | 0.55 |
| Matsuda S_I_ | 9.12±5.72 | 9.26±5.15 | 11.34±8.53 | 0.13 |

**Table S9** Anthropometric and quantitative metabolic characteristics of 5,645 middle-aged Danish Inter99 participants stratified according to the *AGPAT6* rs11785763 genotype

| *AGPAT6* rs11785763 | AA | GA | GG | p value |
| --- | --- | --- | --- | --- |
| N (men/women) | 4083(2018/2065) | 1330(636/694) | 118(61/57) |  |
| Age (years) | 45.9±7.8 | 46.1±8.0 | 45.9±7.3 |  |
| Waist (cm) | 86.0±12.7 | 85.6±13.2 | 88.3±14.2 | 0.94 |
| BMI (kg/m^2^) | 26.1±4.4 | 25.9±4.3 | 26.9±4.5 | 0.66 |
| Serum Triglycerides (mmol/l) | 1.3±1.3 | 1.3±1.4 | 1.2±0.6 | 0.22 |
| Serum Total cholesterol (mmol/l) | 5.5±1.1 | 5.5±1.1 | 5.5±1.0 | 0.053 |
| Serum HDL cholesterol (mmol/l) | 1.4±0.4 | 1.4±0.4 | 1.5±0.4 | 0.50 |
| **Plasma glucose** (mmol/l) |  |  |  |  |
| Fasting | 5.46±0.50 | 5.44±0.54 | 5.54±0.53 | 0.85 |
| 120 min | 5.97±1.56 | 5.89±1.47 | 5.99±1.68 | 0.20 |
| **Serum insulin** (pmol/l) |  |  |  |  |
| Fasting | 41±26 | 40±25 | 46±31 | 0.39 |
| 120 min | 208±204 | 200±167 | 228±219 | 0.85 |
| **Insulin secretion indices** |  |  |  |  |
| Insulinogenic index | 30.0±19.8 | 29.0±17.7 | 34.1±28.8 | 0.93 |
| BIGTT-AIR | 1876±1124 | 1816±979 | 1921±932 | 0.39 |
| **Insulin sensitivity indices** |  |  |  |  |
| HOMA-IR (pmol*mmol/l) | 10.02±6.87 | 9.93±6.59 | 11.33±7.91 | 0.44 |
| BIGTT-S_I_ | 9.40±3.95 | 9.59±3.90 | 8.60±4.25 | 0.63 |
| Stumvoll S_I_ | 0.10±0.02 | 0.10±0.02 | 0.10±0.02 | 0.78 |
| Matsuda S_I_ | 9.16±5.85 | 9.11±5.20 | 8.26±5.28 | 0.52 |

**Table S10** Anthropometric and quantitative metabolic characteristics of 5,645 middle-aged Danish Inter99 participants stratified according to the *AGPAT6* rs7357415 genotype

| *AGPAT6* rs7357415 | AA | GA | GG | p value |
| --- | --- | --- | --- | --- |
| N (men/women) | 3381(1672/1709) | 1861(910/951) | 276(124/152) |  |
| Age (years) | 45.9±7.9 | 46.1±7.7 | 46.4±7.7 |  |
| Waist (cm) | 86.1±12.8 | 85.8±13.0 | 85.5±12.0 | 0.59 |
| BMI (kg/m^2^) | 26.1±4.5 | 26.0±4.2 | 26.0±4.3 | 0.63 |
| Serum Triglycerides (mmol/l) | 1.3±1.5 | 1.3±1.1 | 1.2±1.0 | 0.81 |
| Serum Total cholesterol (mmol/l) | 5.5±1.1 | 5.5±1.1 | 5.5±1.0 | 0.89 |
| Serum HDL cholesterol (mmol/l) | 1.4±0.4 | 1.4±0.4 | 1.5±0.4 | 0.88 |
| **Plasma glucose** (mmol/l) |  |  |  |  |
| Fasting | 5.46±0.51 | 5.45±0.52 | 5.44±0.47 | 0.63 |
| 120 min | 5.97±1.55 | 5.93±1.52 | 5.97±1.56 | 0.43 |
| **Serum insulin** (pmol/l) |  |  |  |  |
| Fasting | 41±26 | 41±26 | 40±23 | 0.85 |
| 120 min | 209±203 | 201±188 | 212±207 | 0.82 |
| **Insulin secretion indices** |  |  |  |  |
| Insulinogenic index | 29.5±19.4 | 30.0±19.8 | 30.9±19.2 | 0.084 |
| BIGTT-AIR | 1866±1134 | 1848±1012 | 1875±1042 | 0.71 |
| **Insulin sensitivity indices** |  |  |  |  |
| HOMA-IR (pmol*mmol/l) | 10.00±6.83 | 10.00±6.88 | 9.74±5.88 | 0.91 |
| BIGTT-S_I_ | 9.44±4.00 | 9.46±3.83 | 9.46±3.86 | 0.34 |
| Stumvoll S_I_ | 0.10±0.02 | 0.10±0.02 | 0.10±0.02 | 0.14 |
| Matsuda S_I_ | 9.19±5.78 | 9.03±5.30 | 9.26±6.59 | 0.95 |

**Table S11** Anthropometric and quantitative metabolic characteristics of 5,645 middle-aged Danish Inter99 participants stratified according to the *AGPAT6* rs10504041 genotype

| *AGPAT6* rs10504041 | AA | GA | GG | p value |
| --- | --- | --- | --- | --- |
| N (men/women) | 3586(1787/1799) | 1705(805/900) | 225(116/109) |  |
| Age (years) | 45.8±7.9 | 46.2±7.7 | 46.0±8.0 |  |
| Waist (cm) | 85.9±12.9 | 86.0±12.9 | 84.8±11.6 | 0.71 |
| BMI (kg/m^2^) | 26.0±4.5 | 26.1±4.3 | 25.7±4.0 | 0.99 |
| Serum Triglycerides (mmol/l) | 1.3±1.5 | 1.3±1.0 | 1.3±1.0 | 0.86 |
| Serum Total cholesterol (mmol/l) | 5.5±1.1 | 5.6±1.1 | 5.5±1.0 | 0.33 |
| Serum HDL cholesterol (mmol/l) | 1.4±0.4 | 1.5±0.4 | 1.4±0.4 | 0.56 |
| **Plasma glucose** (mmol/l) |  |  |  |  |
| Fasting | 5.45±0.51 | 5.45±0.50 | 5.44±0.51 | 0.67 |
| 120 min | 5.96±1.57 | 5.94±1.50 | 5.78±1.44 | 0.17 |
| **Serum insulin** (pmol/l) |  |  |  |  |
| Fasting | 41±26 | 41±26 | 39±23 | 0.89 |
| 120 min | 210±200 | 202±196 | 183±141 | 0.12 |
| **Insulin secretion indices** |  |  |  |  |
| Insulinogenic index | 29.7±19.3 | 29.9±20.0 | 31.1±19.9 | 0.30 |
| BIGTT-AIR | 1860±1089 | 1856±1082 | 1878±944 | 0.60 |
| **Insulin sensitivity indices** |  |  |  |  |
| HOMA-IR (pmol*mmol/l) | 10.02±6.81 | 10.04±6.92 | 9.49±6.10 | 0.86 |
| BIGTT-S_I_ | 9.43±4.02 | 9.48±3.85 | 9.65±3.67 | 0.076 |
| Stumvoll S_I_ | 0.10±0.02 | 0.10±0.02 | 0.10±0.02 | 0.022 |
| Matsuda S_I_ | 9.18±5.80 | 9.02±5.38 | 9.57±6.18 | 0.52 |
